# Bone marrow ectopic expression of a non-coding RNA in childhood T-cell acute lymphoblastic leukemia with a novel t(2;11)(q11.2;p15.1) translocation

**DOI:** 10.1186/1476-4598-7-80

**Published:** 2008-10-23

**Authors:** Maria Corsignano Guastadisegni, Angelo Lonoce, Luciana Impera, Francesco Albano, Pietro D'Addabbo, Sebastiano Caruso, Isabella Vasta, Ioannis Panagopoulos, Anna Leszl, Giuseppe Basso, Mariano Rocchi, Clelia Tiziana Storlazzi

**Affiliations:** 1 Department of Genetics and Microbiology, University of Bari, Bari, Italy; 2 Department of Hematology, University of Bari, Bari, Italy; 3 Laboratorio di Genetica Medica, AUSL LE, P.O. "Vito Fazzi", Lecce, Italy; 4 Oncoematologia Pediatrica, AUSL LE, P.O. "Vito Fazzi", Lecce, Italy; 5 Department of Clinical Genetics, University Hospital, Lund, Sweden; 6 Department of Pediatrics, Laboratory of Pediatric Onco-Hematology, University of Padova, Padova, Italy

## Abstract

Chromosomal translocations play a crucial role in tumorigenesis, often resulting in the formation of chimeric genes or in gene deregulation through position effects. T-cell acute lymphoblastic leukemia (T-ALL) is associated with a large number of such rearrangements. We report the ectopic expression of the 3' portion of EST *DA926692 *in the bone marrow of a childhood T-ALL case showing a t(2;11)(q11.2;p15.1) translocation as the sole chromosome abnormality. The breakpoints, defined at the sequence level, mapped within *HPS5 *(Hermansky Pudlak syndrome 5) intron 1 at 11p15.1, and *DA926692 *exon 2 at 2q11.2. The translocation was accompanied by a submicroscopic inversion that brought the two genes into the same transcriptional orientation. No chimeric trancript was detected. Interestingly, Real-Time Quantitative (RQ)-PCR detected, in the patient's bone marrow, expression of a 173 bp product corresponding to the 3' portion of *DA926692*. Samples from four T-ALL cases with a normal karyotype and normal bone marrow used as controls were negative. It might be speculated that the juxtaposition of this genomic segment to the CpG island located upstream *HPS5 *activated *DA92669 *expression. RQ-PCR analysis showed expression positivity in 6 of 23 human tissues examined. Bioinformatic analysis excluded that this small non-coding RNA is a precursor of micro-RNA, although it is conceivable that it has a different, yet unknown, functional role. To the best of our knowledge, this is the first report, in cancer, of the activation of a small non-coding RNA as a result of a chromosomal translocation.

## Findings

Acquired balanced chromosomal translocations provide crucial diagnostic and prognostic data in cancer patients. They are probably pathogenetically significant as initiating events if present as the only cytogenetic changes [1]. These rearrangements often result in the formation of a fusion gene, encoding a chimeric oncogenic protein, or in the deregulation of the expression pattern of genes flanking the breakpoint regions by promoter swapping [1-3]. Overall, gene deregulation can be accomplished through position effects, such as a gene juxtaposition close to an active chromatin domain. More rarely, translocations may result in gene downregulation because of hypermethylation of CpG islands at the breakpoint [4], or in the formation of chimeric transcripts that do not encode for a protein with oncogenic potential [5].

T-ALL, as other hematological malignancies, is associated with a number of chromosomal abnormalities, resulting either in a position effect or in a gene fusion [6].

Here we report the cloning of a novel balanced t(2;11)(q11.2;p15.1) translocation, found as the sole cytogenetic abnormality in the BM of a childhood T-ALL case, accompanied by the ectopic expression of the 3' portion of EST *DA926692*.

A 14-year-old male patient was referred to our hospital for rhinolalia. Total body CT disclosed a diffuse enlargement of the rhinopharynx vault, lymphoid hyperplasia of the Waldeyer's tonsillar ring, and a mediastinal enlargement. BM aspiration showed 90% of blast cells. Flow cytometry revealed that blast cell population was positive for CD 45, 2, 5, 7, and cytoplasmic CD3, while negative for CD1a and surface CD3. These findings were consistent with a diagnosis of early T-ALL, thus the patient started treatment based on AIEOP-BFM ALL 2000 protocol [7].

At the end of induction therapy, the patient was categorized as "high risk" due to incomplete remission, as per protocol stratification criteria. After consolidation therapy, the BM aspirate showed no haematological remission, so treatment continued following the AIEOP LLA REC 2003 protocol and complete hematological remission was obtained.

One year from diagnosis, the patient underwent HLA-identical hematopoietic stem cell transplantation from an unrelated donor. He is now doing well and is still in hematologic remission.

**Figure 1:**
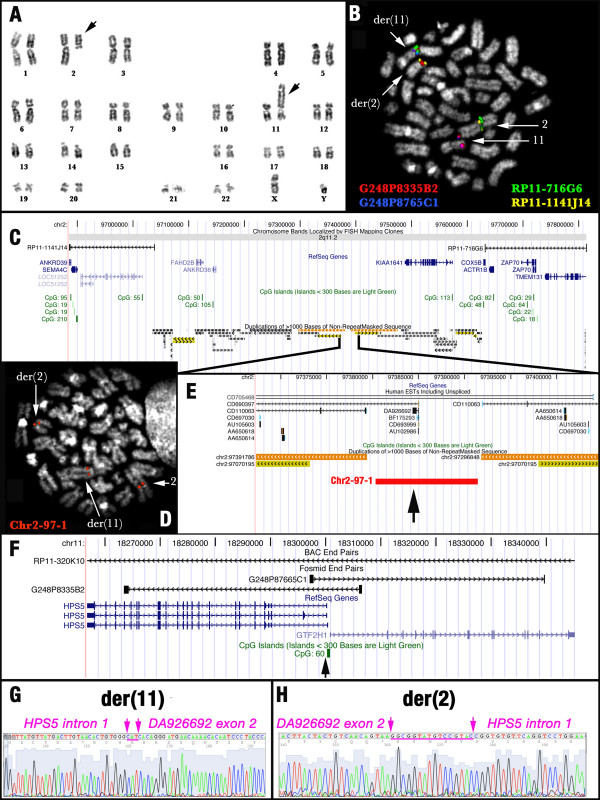
**Results of the cytogenetic and genomic characterization of the t(2;11) traslocation breakpoints**: A) Karyotype of the case described in the present study. The black arrows point on derivative chromosomes 2 and 11. B) FISH results obtained with fosmids and BAC clones delimiting the breakpoint regions on der(2) and der(11). C) Map of chromosome 2 breakpoint region, according to the latest release of the UCSC Genome Browser (March 2006), delimited by BAC clones RP11-1141J14 (left) and RP11-716G6 (right). RefSeq Genes and CpG islands are represented in blue and green, respectively. Intra-chromosomal duplications are reported at the bottom of the figure. Gray, yellow and orange colours refer to the percentage of sequence similarity of the duplication (respectively 90–98%, 98–99%, and >99%). D) FISH results obtained using the Chr2-97-1 Long-PCR product on the bone marrow of the patient. E) Detailed magnified map of the chromosome 2 breakpoint region, showing the location of the Chr2-97-1 probe. F) Map of the breakpoint region on chromosome 11. G) and H) Partial chromatograms of the junctions sequences on both derivative chromosomes 11 (G) and 2 (H). Inserted nucleotides [three (CAT) and sixteen (GGCGGTATGTCCGTAC) at der(11) and der(2), respectively] at the junctions are underlined in purple.

G-banding analysis of bone marrow metaphases at onset revealed the karyotype 46,XY,t(2;11)(q11.2;p15.1) [27] [8] (Fig. 1A). FISH analysis excluded the presence of known rearrangements (data not shown), and revealed that the chromosome 11 breakpoint was encompassed by the BAC clone RP11-320K10 (chr11:18,229,291–18,413,758) and the fosmid G248P8335B2 (Fig. 1B), harboring the 5' UTR regions of two genes, *HPS5 *and *GTF2H1*, located 422 bp apart and in opposite transcriptional orientation (Fig. 1F).

The break on chromosome 2 was mapped within the interval chr2:96,884,868–97,812,993, delimited by the BACs RP11-1141J14 (chr2:96,884,868–97,038,565) and RP11-716G6 (chr2:97,631,541–97,812,993) at 2q11.2, yielding FISH signals on der(2) and der(11), respectively (Figs. 1B,C). It was not possible to further refine this breakpoint region by large-insert probes, as it is almost entirely composed of segmental duplications. We identified two small regions devoid of duplicons: chr2:97,284,347–97,296,849 (12,503 bp) and chr2:97,379,383–97,391,787 (12,405 bp) (Figure 1E). We obtained a long-range PCR product from the latter interval, that appeared to encompass the breakpoint on chromosome 2 (Figure 1D,E). This conclusion, however, was regarded with caution, since the region is rich in copy number variants (UCSC, Structural Variation track) providing a possible alternative explanation for the FISH results. The amplified interval does not contain known genes, although a number of ESTs of unknown function are annotated (Fig. 1E).

Vectorette-PCR using a reverse primer (hpsgtf-R, Table 1), specific for the genomic segment between genes *HPS5 *and *GTF2H1*, produced a fragment of approximately 800 bp. The sequence (Accession No. EU617360) showed that chromosome 11 at nt 18,300,006 (*HPS5 *intron 1) was fused with chromosome 2 at nt 97,384,504 (EST *DA926692 *exon 2) (Fig. 1G). Unexpectedly, sequences from chromosomes 2 and 11 had the same orientation.

**Table 1:** Primers for PCR and sequencing.

**Designation^a^**	**Sequence (5'->3')**	**Position^b^**	**Gene**
chr2-97-1F	AGCCTGTGGTGTGTGTATGAAC	chr2:97,380,441–97,380,462	-
chr2-97-1R	TGAGTGTAGGTGACTGGTGAGG	chr2: 97,391,465–97,391,486	-
hpsgtf-R	TGACACCTTCCGCTAGTTCC	chr11:18,300,606–18,300,625	-
Hps5-F	CCTCCCTGCTTCTTTTTCCA	chr11:18,299,339–18,299,358	*HPS5*
HPS5ex20-3216F^c^	TGCAGGTCTTGTGGTTTCTG	chr11:18,263,475	*HPS5*
HPS5ex21-3255R^c^	TCTTCTCTCCAGCTCCAAACA	chr11:18,261,979–18,261,999	*HPS*
HPS5ex1-19F^c^	TTCACGTTCCGCTCTTAGTG	chr11:18,300,260–18,300,279	*HPS5*
HPS5ex1-96R^c^	CACATCCAGGGCAGTACCTC	chr11:18,300,183–18,300,202	*HPS5*
HPS5ex1-181F	TGGTTACTGGGTCTCCTCTCA	chr11:18,300,107–18,300,127	*HPS5*
HPS5-intr1-F	TGTGTTTTGTTCATCCCTGTG	chr11:18,300,006–18,300,026	*HPS5*
DA926692 ex1F^c^	TCAGTCCCAGTCAGGACACA	chr2:97,384,972–97,384,991	*DA926692*
DA926692 ex1R^c^	AGAGCCAGAGCAGCAGGAG	chr2:97,384,918–97,384,936	*DA926692*
DA926692 ex2aF	CCATCAAGGGAAGCAGATGT	chr2:97,384,457–97,384,476	*DA926692*
DA926692 ex2aR	GAGGCACCAGGAGAAGCAT	chr2:97,384,418–97,384,436	*DA926692*
DA926692ex2anewF^c^	CCCACAGTGTTACAAGTCATAACATA	chr2:97,384,479–97,384,504	*DA926692*
DA926692ex2anewR^c^	AGGAAGCTTCATGGCTCCTT	chr2:97,384,334–97,384,353	*DA926692*
Beta-act F^c^	CTGGAACGGTGAAGGTGACA	chr7:5,533,739–5,533,758	*ACTB*
Beta-act R^c^	AAGGGACTTCCTGTAACAACGCA	chr7:5,533,619–5,533,641	*ACTB*
CD110063-R	CACCAGGAGGCAGACGAG	chr2:97,391,951–97,391,968	*CD110063*
ACTB-F	GGCATCGTGATGGACTCCG	chr7:5,534,773–5,534,789	*ACTB*
ACTB-R	GCTGGAAGGTGGACAGCGA	chr7:5,533,972–5,533,990	*ACTB*
GTF2H1ex9-1201F	AGCAGTCAAAAGGGCGAAAT	chr11:18,326,030–18,326,043/18,330,004–18,330,010	*GTF2H1*
GTF2H1ex10-1272R	TTCTTGAGGTTTAGTGCAATCG	chr11:18,330,062–18,330,083	*GTF2H1*
ANKex2-351F	CCAACCGGAAATGGTACATC	chr2:97,147,558–97,147,577	*ANKRD36*
ANKex2-451R	AGAGTTGCACAAGCCTCCTG	chr2:97,147,823–97,147,842	*ANKRD36*
FAHD2Bex5-829F	TGTAGCAGATCCACACAACTTAAA	chr2:97,113,730–97,113,742/97,115,163–97,115,171	*FAHD2B*
FAHD2Bex6-864R	GACGACTTCCCCATTCACTC	chr2:97,113,695–97,113,714	*FAHD2B*
KIAA1641ex2-214F	AGAAAATGGGATGCAGGATT	chr2:97,492,508–97,492,527	*KIAA1641*
KIAA1641ex2-245R	GAAATTGACTTCTCATCTGGTCTAA	chr2:97,490,960–97,490,957/chr2:97,492,476–97,492,496	*KIAA1641*
ZAP70ex11-1678F	AGCTACTACACTGCCCGCTCA	chr2:97,720,549–97,720,560/97,720,651–97,720,660	*ZAP70*
ZAP70ex11-1749R	CTGCGGCTGGAGAACTTG	chr2:97,720,709–97,720,726	*ZAP70*

^a ^F denotes forward; R denotes reverse;

^b ^Position, at nucleotide level, according to the UCSC database, using the BLAT tool ;

^c^Primer used in Real-Time PCR analysis.

The same approach was used to clone the reciprocal genomic fusion *DA926692/HPS5*. Vectorette PCR with a forward primer (hps5-F, Table 1) designed within *HPS5 *intron 1 yielded a fragment of 1,050 bp. The sequence (Accession No. EU617359) revealed that chromosome 2, at nt 97,384,507 (*DA926692 *exon 2), was fused with chromosome 11 at nt 18,299,998 (*HPS5 *intron 1) (Fig. 1H). Again, sequences from the two chromosomes had the same orientation. These observations clearly suggested that one of the two sequences had undergone a submicroscopic inversion before translocation. As a consequence, *DA926692 *and *HPS5 *were brought, by the inversion, into the same orientation.

5'- and 3'-RACE-PCR as well as RT-PCR experiments, aimed at cloning possible *HPS5/DA926692 *chimeric transcripts, failed.

**Figure 2:**
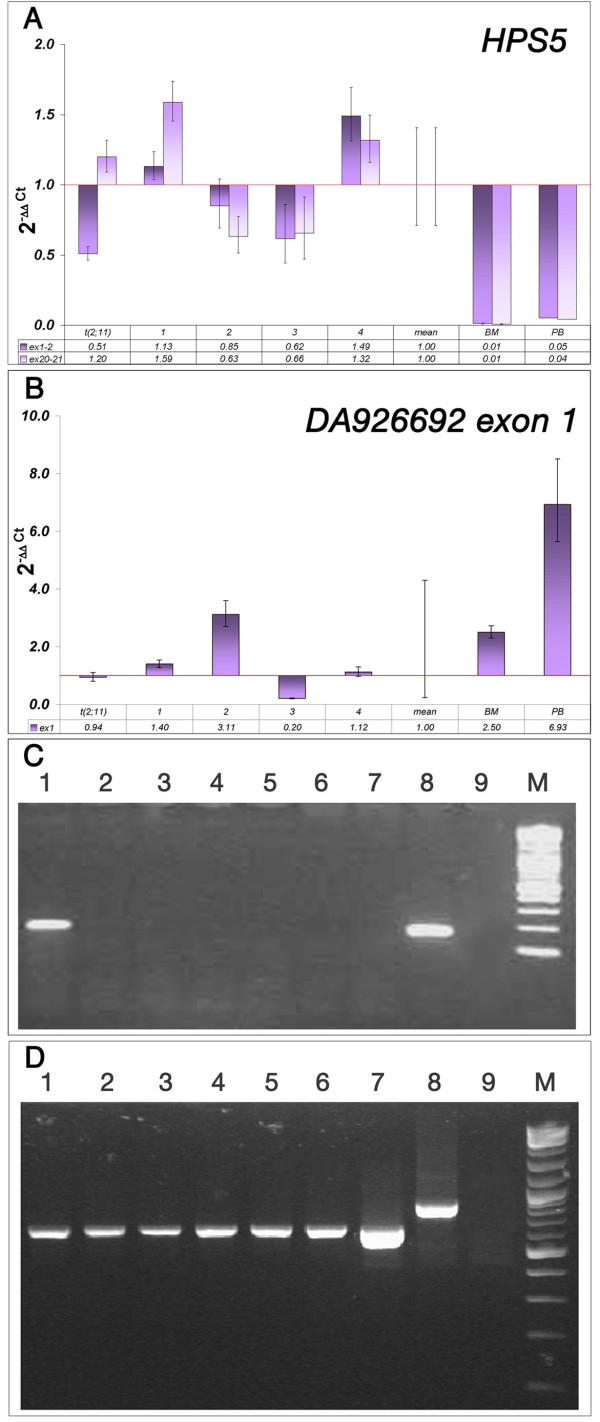
**Results of the *HPS5 *and *DA926692 *gene expression analysis**: A) and B) RQ-PCR analyses of *HPS5*, and *DA926692 *exon1, respectively, in the present case [t(2;11), four control childhood T-ALL samples [immunophenotype: early (#1,2), thymic (#3), and mature (#4) T-ALL], the mean Ct value of the controls (mean), normal BM and PB. A) The results showed no statistically significant change in *HPS5 *expression level with primers for exon1 (HPS5ex1-19F+HPS5ex1-96R) and exons 20-21 (HPS5ex20-3216F and HPS5ex21-3255R), if compared with the mean Ct value of the childhood T-ALL controls. B) The results showed comparable *DA926692 *transcriptional levels, when evaluated with exon 1 primers (DA926692ex2F and DA926692ex2R), between the patient with t(2;11) and the mean Ct value of the controls. C) RT-PCR results obtained with DA926692ex2anewF and DA926692ex2anewR (Table 1), showing a band of 173 bp only in the patient's bone marrow (lane 1) and normal genomic DNA (lane 8). Lanes 2-5 correspond to four control childhood T-ALL bone marrow. Lanes 6, 7 and 9 are normal bone marrow, normal peripheral blood, and blank, respectively. M: 2-Log DNA Ladder (New England Biolabs, Milan, Italy). D) Control RT-PCR with *ACTB *primers to check the RNA quality, excluding contamination of genomic DNA in the patient's bone marrow RNA.

Further RQ-PCR analyses aiming at quantifying the expression levels of genes flanking the breakpoints, i.e. *HPS5 *(Fig. 2A) and *GTF2H1 *(data not shown) on chromosome 11, and *ANKRD36*, *FAHD2B*, *KIAA1641*, *ZAP70 *on chromosome 2, revealed no misexpression in the present case compared to other T-ALL cases without the t(2;11) translocation (data not shown).

The same approach was used for *DA926692*. A primer set specific for exon 1, upstream to the breakpoint, revealed no statistically significant difference in expression level compared to controls (Fig, 2B). Interestingly, a primer pair designed in exon 2 (Table 1), downstream to the breakpoint, detected expression only in the t(2;11) case. RT-PCR with the same primer pair revealed a band of 173 pb only in the patient's BM and in the genomic DNA sample, but neither in the T-ALL controls, nor in normal BM or PB (Fig. 2C). These results were confirmed by nested RT-PCR with the primer pair DA926692 ex2a, further validated by the sequencing of PCR products (data not shown). Purity of all RNA samples was tested using *ACTB *primers in PCR experiments (Fig. 2D).

**Figure 3:**
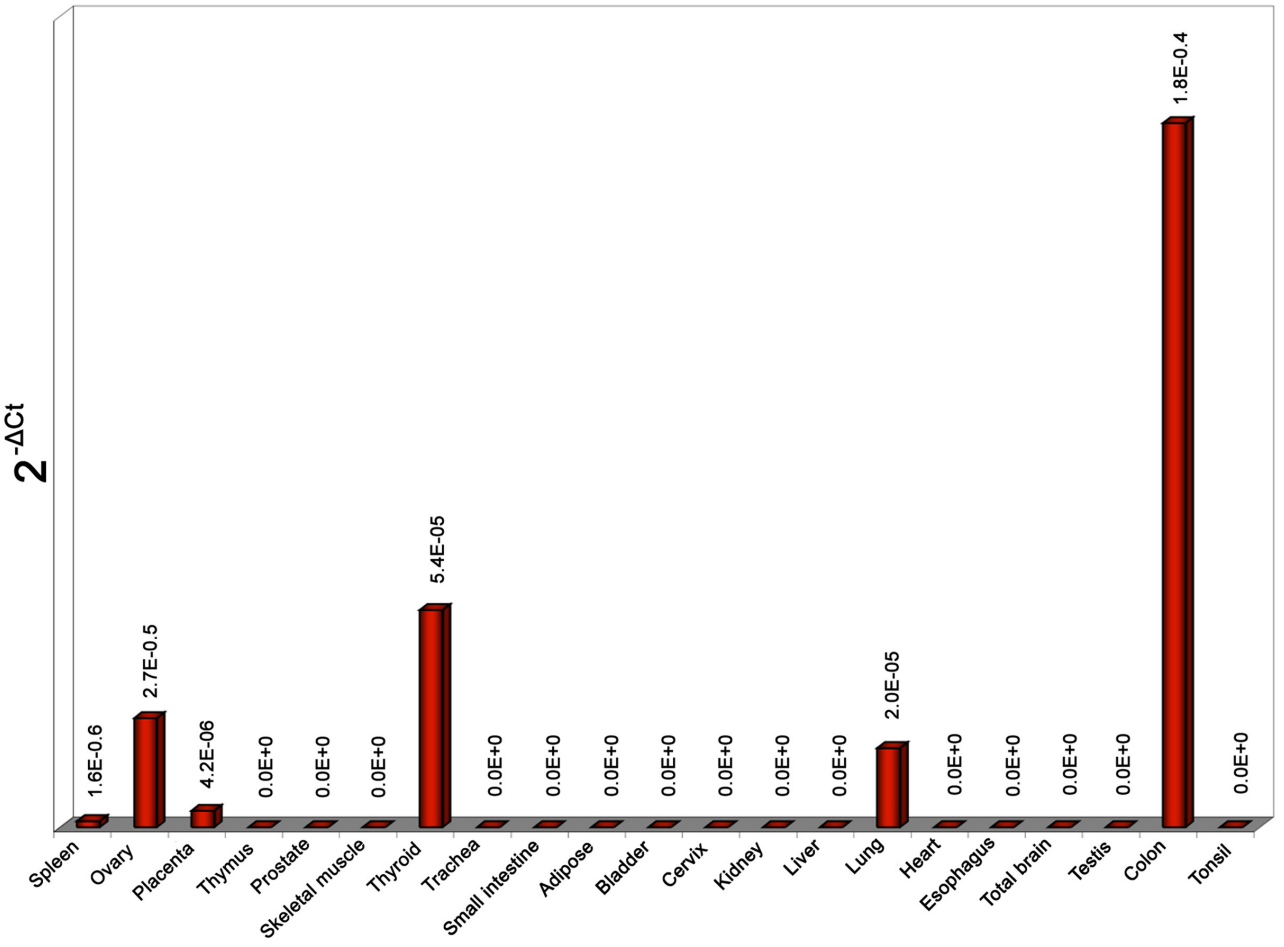
**Tissue expression pattern of EST *DA926692***: *DA926692 *expression analysis was performed using cDNA multiple tissue (Ambion, Milan, Italy) First Choice Total RNA Survey Panel (Catalog No. AM6000) (adipose, bladder, brain, cervix, esophagus, heart, kidney, liver, lung, ovary, placenta, prostate, skeletal muscle, small intestine, spleen, testes, thymus, thyroid, trachea), according to the manufacturer's instructions. We have also tested a pool of tonsil cDNA extracted from three normal individuals. The primer combination used was DA926692ex2anew (F+R). The results, evaluated by RQ-PCR, showed positivity of spleen, ovary, placenta, thyroid, lung, and colon. Compared with the reference *ACTB *gene expression levels (data not shown), the overall *DA926692 *expression in the positive tissues was found to be relatively low (2^-ΔCt ^≤ 1.8 × 10^-4^). The strongest expression was seen in the colon.

Next, we investigated the expression pattern of 3'*DA926692 *by RQ-PCR in 21 adult tissues. The results showed the occurrence of the transcript in 6 of 23 examined tissues (Fig. 3) [normal BM and PB were already shown to be negative (Fig. 2C)].

Notably, *in silico *translation of the sequence corresponding to the 173 bp PCR product showed that it contained a high density of stop codons resulting in very short ORFs.

We hypothesized that this ncRNA could represent a precursor of an miRNA, but the miRNA prediction table produced by miRRim  and a sequence similarity search at miRBase  showed no significant matches (data not shown). Similarly, miRNA predictions by ProMiR II  and miR-abela  did not retrieve any positive results (data not shown).

Finally, we used the Gene Prediction tracks of the UCSC Genome Browser to discover if *DA926692 *might be part of a more complex predicted gene. Notably, the N-SCAN PASA-EST track showed that *DA926692 *and the EST *CD110063*, located in a double position both proximally (chr2:97,362,909–97,379,287) and distally (chr2:97,391,882–97,408,261) to *DA926692 *(Fig. 1E), may be parts of the predicted gene chr2.98.005.a (data not shown).

To verify this hypothesis, we checked for chr2.98.005.a gene expression using the primer combination DA926692ex2anewF+CD110063-R in the patient's BM cDNA. No PCR products were recovered.

In conclusion, we describe a t(2;11) translocation in T-ALL not resulting in any *HPS5/DA926692 *chimeric transcript. The impact on cancer causation of a category of "unproductive" translocations, such as *ETV6 *gene fusions, remains unclear [5]. In such cases, the pathogenetic outcome does not seem to coincide with the formation of a chimeric protein, but perhaps with the production of a truncated form of original proteins (loss of function), or the deregulation of flanking genes.

Here, the only observed gene expression change consisted in the ectopic expression of the 3' portion of *DA926692 *in the patient's BM (Fig. 2C). One hypothesis is that the juxtaposition, at a distance of only 196 nt, of this genomic segment to the CpG island located upstream to both *HPS5 *and *GTF2H1 *(chr11:18,300,202–18,300,814) could have activated its expression in the BM cells of the patient.

*DA926692 *was isolated from a small intestine cDNA library, described as similar to Ig kappa chain V-I region HK103 precursor , but no known function has yet been reported. Its expression in some human adult tissues (Fig. 3) may suggest a potential function of this transcript in the positive samples.

As its function is unclear, it is difficult to speculate on a possible role of this short ncRNA in T-ALL tumorigenesis. As the bioinformatic analysis excluded the possibility that it could behave as a pre-miRNA, we could speculate that this ncRNA may belong to the class of small RNA (20–300 nt), commonly found as transcriptional and translational regulators [9,10]. A variety of complex cellular mechanisms such as gene transcription, chromatin structure dynamics, and others have already been connected to their function [10]. Furthermore, changes in expression levels of ncRNAs have been associated with different types of cancer [9].

In this context, we could speculate that the ectopic expression of the 3' portion of *DA926692 *in chilhood T-ALL could have a pathogenetic role, even if of still unclear significance. It is presently unknown, however, if the described rearrangement represents a primary or a secondary genetic aberration at disease onset. Further studies on similar cases may be extremely important to confirm this new impact of chromosomal translocation, i.e. activation of ncRNA in tumors.

## Abbreviations

(T-ALL): T-cell Acute Lymphoblastic Leukemia; (TCR): T-Cell Receptor; (CT): Computed Tomography; (BM): Bone Marrow; (PB): Peripheral Blood; (FISH): Fluorescence In Situ Hybridization; (BAC): Bacterial Artificial Chromosome; (*HPS5*): Hermansky-Pudlak syndrome 5 isoform b; (*GTF2H1*): general transcription factor IIH, polypeptide 1; (nt): nucleotide; (RQ-PCR): Real-Time Quantitative PCR; (*ANKRD36*): ankyrin repeat domain 36; (*FAHD2B*): fumarylacetoacetate hydrolase domain containing; (*KIAA1641*): hypothetical protein LOC57730; (*ZAP70*): zeta-chain associated protein kinase 70 KDa; (ORFs): Open Reading Frames; (miRNAs): microRNAs; (ncRNA): non-coding RNA.

## Competing interests

The authors declare that they have no competing interests.

## Authors' contributions

MCG and AL designed research and performed molecular analyses. FA and IV contributed in clinical data collection and analysis. SC and AL performed classical cytogenetic analysis and contributed in the development of the study. LI performed molecular cytogenetic analysis. PD performed bioinformatic analysis. IP contributed in molecular data analysis and interpretation of the results. MR and GB gave final approval to the manuscript. CTS supervised the study and drafted the manuscript.
